# Deep Brain Stimulation for Refractory Temporal Lobe Epilepsy. Current Status and Future Trends

**DOI:** 10.3389/fneur.2022.796846

**Published:** 2022-02-23

**Authors:** Francisco Velasco, Pablo E. Saucedo-Alvarado, Daruny Vazquez-Barron, David Trejo, Ana Luisa Velasco

**Affiliations:** Unit for Stereotactic and Functional Neurosurgery, Epilepsy Clinic, Mexico General Hospital “Dr. Eduardo Liceaga”, Mexico City, Mexico

**Keywords:** deep brain stimulation, refractory mesial temporal lobe epilepsy, hippocampal sclerosis, para-hippocampal cortex, subiculum, neuropsychological evaluation, GABAergic antiepileptic mechanisms

## Abstract

A comparative analysis of the targets for deep brain stimulation (DBS) to treat refractory temporal lobe epilepsy and the rationale for its use is presented, with an emphasis on the latency to obtain the significant antiepileptic effect and the long-term seizure control. The analysis includes consideration of surgical techniques currently used to optimize antiseizure effects and decrease surgical risks. Seizure control is similar for programed DBS and DBS responsive to abnormal cortical or subcortical electroencephalogram (EEG) activity. There is no difference in the long-term seizure control between programmed and responsive and intermittent or continuous DBS. However, intermittent programed DBS may have a significant antiseizure effect starting in the first month when applied to a non-sclerotic tissue such as the parahippocampal cortex. DBS induces no neuropsychological deterioration.

## Introduction

The rationale for performing deep brain stimulation (DBS) as a treatment for refractory seizures originating from the temporal lobe was provided by previous reports: subacute electrical stimulation (SACS) through electrodes used to define the seizure onset zone (SOZ) decreased the interictal spikes, increased threshold for post-discharges, and reduced the incidence of occurring spontaneous seizures (1). SACS induced a profound decrease in the local cerebral blood flow of the stimulated site evidenced by a single-photon emission computerized tomography (SPECT) scan. Surgical specimens of patients subjected to SACS demonstrated an increase in the expression of benzodiazepine receptors in the hippocampus and even more in the parahippocampal cortex compared to specimens of patients with epilepsy that had not received SACS (2). Also, gamma-aminobutyric (GABA) increased in the specimens subjected to SACS (3). These observations suggested that DBS may have a significant GABA-mediated inhibitory effect in the epileptogenesis of drug-resistant epilepsy (DRE). A series of patients subjected to long-term DBS confirmed a significant antiepileptic impact when DBS was applied in the hippocampus (H), particularly, in patients without evidence of hippocampal sclerosis by MRI (4, 5). The conclusion of all these reports was that DBS represented an alternative to treat patients with DRE originated in the medial temporal lobe, particularly those without hippocampal sclerosis (HS), with seizures initiated in the dominant hemisphere for language and memory, or patients with bilateral independent foci in whom resective surgery may induce cognitive deficits. For patients with HS, sequential protocols were designed to study the antiepileptic effect of DBS in anatomical structures that have been reported related to the genesis or propagation of interictal activity in temporal lobe epilepsy (6–8).

## Study Design

This study is a retrospective analysis of the data collected from three pilot studies of Temporal Lobe Drug-Resistant Epilepsy (DRE) in patients with HS ipsilateral to the SOZ and treated by DBS in the H (*n* = 4) (4), subiculum (S) (*n* = 6) (8), and parahippocampal cortex (PHC) (*n* = 6) (9). The patients enrolled had bilateral independent epileptic foci detected by Stereo-electroencephalographic (SEEG) recordings, patients with an epileptic focus in the dominant hemisphere with high risk for ablative surgery for memory or speech deterioration, and patients that decided for a less invasive surgical treatment. All patients underwent protocol for epilepsy surgery that included 3–4 months of the baseline period, SEEG recordings for (SOZ) localization, implantation of DBS systems, 3–8 months double-blind (ON and OFF) stimulation periods, and 18 months follow-up ON stimulation. A neuropsychological test battery was applied before and at the end of the study.

## Baseline Data

The baseline period included an accurate description of seizure numbers of Focal Impaired Aware Seizures (FIAS) and Focal Evolved to Bilateral Tonic-Clonic seizures (FBTC); EEG confirming paroxysmal activity in the temporal leads; 1.5-T MRI oriented along the hippocampal axis for axial and coronal views confirming the presence of HS; neuropsychological evaluation, including language dominance, through the use of the Spanish version of the dichotic listening test, as well as a test battery to evaluate attention and memory (NEUROPSI), validated for the Spanish-speaking population (10).

## Electrode Implantation and Stimulation Parameters

**Figure 1:**
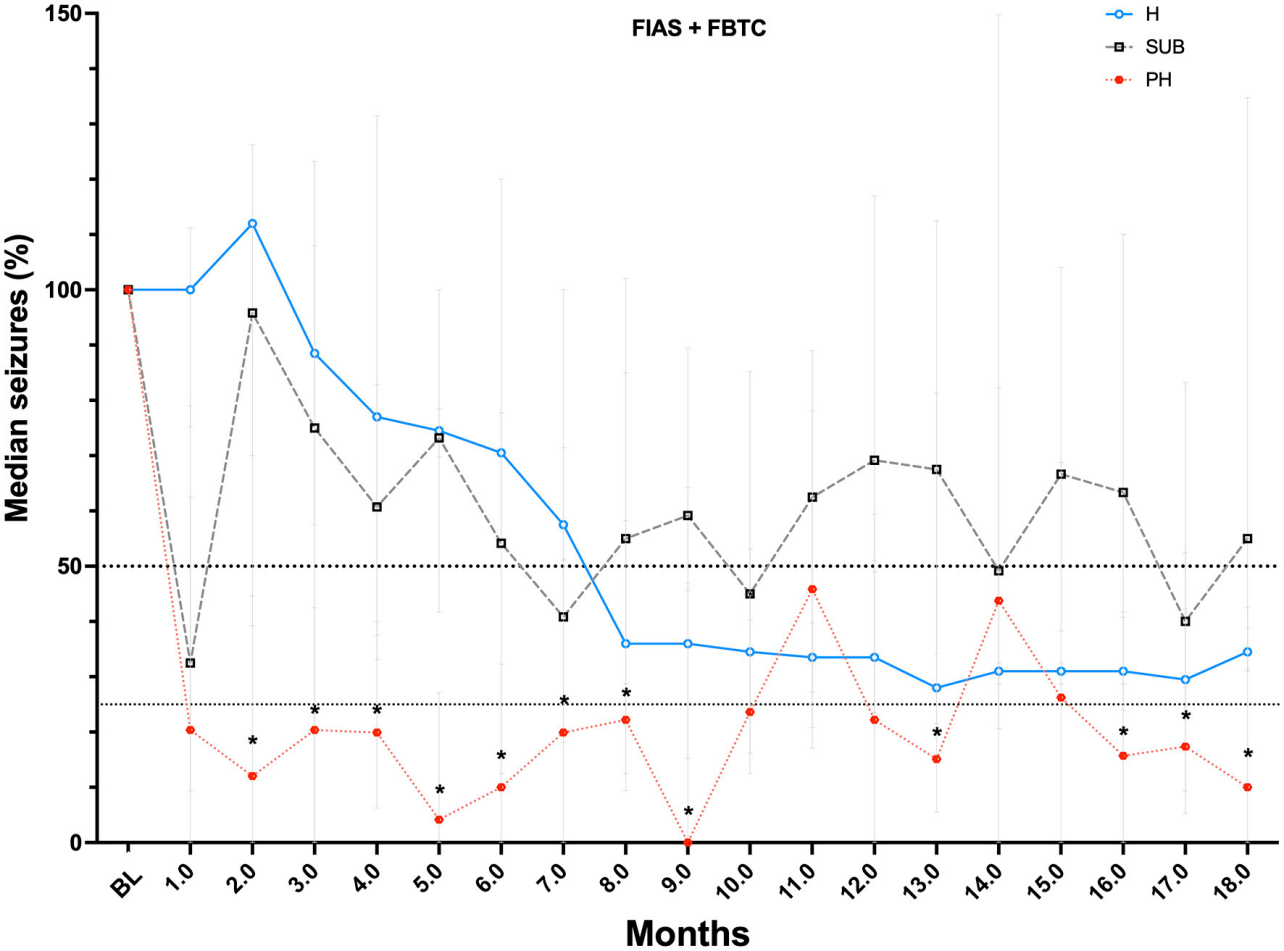
Percentage decrease of seizures from baseline along the 18th month follow-up of the 3 groups studied: Blue line H-DBS, Black line S-DBS and Red line PHC-DBS. * Indicates the significance of seizure reduction *p* < 0.05 for PHC-DBS occurring since the first month on DBS.

On the day of surgery, MRI studies were fused to a CT scan performed with a stereotactic frame in place (Z-D Leibinger, Freiburg, Germany), and trajectories were planned to use the 3A Plus Praezis software (Heidelberg, Germany), aiming at the targets (H, S, or PHC). Since the PHC was a larger target to perform SEEG and subsequent DBS, the electrode implantation for SEEG was guided by PET-CT scans, using the 18 Fluor-Flumazenil (18-FFMZ) as a radiotracer, where the area with the lowest uptake represents the area where the highest concentration of GABA (11). Anti-epileptic drugs (AEDs) were taken OFF during SEEG for the localization of SOZ. For H and S targets, SEEG was performed through intracranial tubular octopolar electrodes (AD-TECH Medical, Racine WI, USA) that were replaced by 3,387 Medtronic DBS electrodes after SOZ localization. For the PHC, 3,391 Medtronic Inc. electrodes (Minneapolis MN), with a center-to-center contact distance of 7.0 mm, were used for both SEEG and DBS, decreasing the number of interventions into a single intracranial procedure. Electrodes were stereotactically placed along the major axis of the hippocampus through occipital burr-holes (Figure 1). SOZ was localized by recording at least 2 spontaneous seizures.

Bipolar stimulation was performed through contiguous contacts in the area where SEEG had been localized, having the more anterior contact(s) at the hippocampus-amygdala junction, acting as cathode and the posterior as the anode. Bilateral DBS was applied in cases where SEEG had demonstrated bilateral independent SOZ. Stimulation parameters were 130 Hz, 450 μs pulse amplitude, 2–3 V, and cyclic 1-min ON and 4-min OFF stimulation.

## Follow-Up

In the three groups, seizure data was obtained from the diaries of seizure occurrence, describing seizure type and frequency, starting 3–4 months before the surgical procedure (Baseline). These diaries were used to report the monthly occurrence of FIAS and FBTC. Focal Aware Seizures (FAS) were considered highly subjective and often not correlated with EEG abnormalities. The three groups maintained AEDs without modifications along with the follow-up.

## Statistical Analysis

All seizures were normalized to a median seizure percentage depending on their baseline seizure frequency. We applied a non-parametrical analysis; first, we used a Wilcoxon test to compare the baseline median seizure percentage with the months of the follow-up and a Mann-Whitney *U*-test between groups.

## Results

Data from 16 patients were collected, 8 males and 8 females. The patients' ages ranged from 18 to 52 years (mean = 29.5, SD = 10.6 years). The baseline seizure median per month was 11 (IQR = 9–33). All patients had HS on MRI corresponding with the side of SOZ, 11 with unilateral HS (6 left, 5 right), five with bilateral HS. SEEG studies demonstrated unilateral SOZ in 11 patients (6 right, 6 left) and bilateral in 4 (Table 1).

**Table 1:** Demography of cases of temporal lobe epilepsy and hippocampus sclerosis treated with deep brain stimulation (DBS) in the hippocampus (H), Subiculum (s), and para-hippocampal cortex (PHC).

	**Age (years)**	**Gender**	**BL seizure median**	**HS**	**SOZ**	**Follow-up**
H-DBS (*n* = 4)	31; SD = ± 13.2	3 M; 1 F	32; IQR = 23–62	L = 3; B = 1	L = 1; R = 2; B = 1	No DB 18mo H-DBS
S-DBS (*n* = 6)	26.7; SD = ± 8.9	4 M; 2 F	5; IQR = 5–12	L = 2; R = 4	L = 3; R = 1; B = 2	DB: 6mo 18mo S-DBS
PHC-DBS (*n* = 6)	23; SD = ± 6	1 M; 5F	8; IQR = 7–19	L = 1; R = 1; B = 4	L = 2; R = 2; B = 2	DB: 8mo 18mo PHC-DBS

*HS, Hippocampal Sclerosis; SOZ, seizure onset zone; mo: months; F, Female; M, Male; H-DBS, hippocampal neuromodulation; S-DBS, subiculum neuromodulation; PHC-DBS, parahippocampal neuromodulation; N, Sample; SD, Standard deviation; IQR, interquartile range; DB, Double-Blind; L, left; R, right; B, bilateral*.

## Seizure Outcome

An insertional effect was the rule in the 3 groups of patients studied from 1 to 2 months. In the PHC-DBS group, there was a decrease in the number of seizures >50% since the first month on DBS (*p* < 0.05), with a median of residual seizures of 20.37% (IQR= 0–62.4%) that was maintained along with the follow-up, except for the fifth month when one patient discontinued AEDs and another presented febrile seizures associated with pneumonia, to end up with a median of residual seizures of 12.04% (IQ = 0–44.5%) (*p* = 0.032). In contrast, S-DBS had an increase of seizures after a month with an insertional effect back to the Baseline (BL) level. The decrease ranged from 40 to 55% but did not reach significance. H-DBS had a progressive decrease of seizure number with a 50% improvement at the eighth month on stimulation (median = 64%, IQ = 42–72%) and remained between 54 and 60% along the following months (Figure 1). Median number of residual seizures at month 18 was 10% (IQR 0–38%) for PHC-DBS, 34.5% (IQR = 35–42.5%) for H-DBS, and 55% (IQR = 35–134%) for S-DBS (Figure 1 and Table 2).

**Table 2:** Comparative seizure outcome and complications of patients with hippocampal sclerosis treated by DBS in the H, S, PHC.

	**Type of seizure**	**6mo seizure reduction (%)**	**12mo seizure reduction (%)**	**18mo seizure reduction (%)**
H-DBS (*n* = 4)	FIAS+FBTC	30; IQR = 23–45	67; IQR = 49–77	66; IQR = 58–69
S-DBS (*n* = 6)	FIAS+FBTC	46; IQR = −20–88	31; IQR = −17–52	45; IQR = −34–69
PHC-DBS (*n* = 6)	FIAS+FBTC	90; IQR = 68–100	78; IQR = 41–100	90; IQR = 62–100

*mo, month; H-DBS, hippocampal neuromodulation; S-DBS, subiculum neuromodulation; PHC-DBS, parahippocampal neuromodulation; N, Sample; FIAS, focal initiated seizures with impaired awareness; FBTC, focal initiated seizures with bilateral tonic clonic seizures; IQR, interquartile range*.

## Neuropsychological Evaluation

All patients in the 3 groups had a suboptimal performance in the NEUROPSI test battery in the preoperative, evaluating memory, and executive functions; some were considerably more deteriorated than others, probably secondary to seizure occurrence and AEDs side effects, and HS. There were no significant changes in the postoperative evaluation; however, all tended to improve in functions related to the temporal lobe (memory) and frontal lobe (executive), and some with better preoperative performance in the 3 groups evolved from mild alteration level to normal level. This was accompanied for re-integration from partial employment to full employment jobs in 3/6 patients and back to school in 1 for the S-DBS group: full employment in 2 and back to school in 1 for the PHC-DBS group.

## Surgical Complications

One patient had skin erosion and local infection on the site of the Internal Pulse Generator (IPG) and was eliminated and replaced in the protocol. One patient with PHC-DBS reported intermittent paresthesia on the territory of the V2 trigeminal branch when the pulse amplitude was above 3.0 V, probably by the current spreading to the trigeminal fossae, which limited DBS programing.

## Discussion

We considered this a pilot study to determine the status of DBS in the temporal lobe structures for the treatment of temporal lobe seizures and explore future trends for improving the safety and efficacy of the procedure. The main drawback of this report is the reduced number of patients studied. In the present analysis, focal “seizures” without impaired awareness (FAS) were not considered in the seizure count since they are highly subjective. Some patients with increment in FAS had extended EEG recordings that disclosed no electroencephalographic correlation between FAS and EEG abnormalities. Besides, most publications on DBS for the treatment of temporal lobe seizures report only incapacitating FIAS and FBTC seizures (12, 13, 15–17, 19, 21).

**Figure 2:**
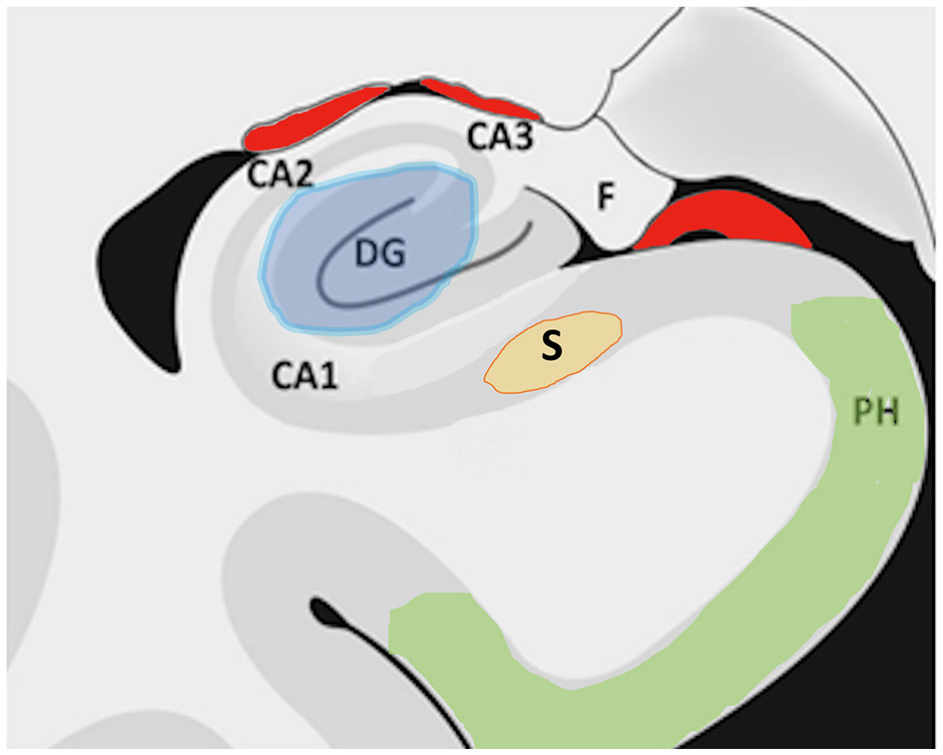
Diagram of a coronal section of the temporal lobe that illustrates the size and location of the studied targets: in blue the hippocampus including dentate gyrus (DG) and Ammon fields C1–C3; subiculum (S); para-hippocampal cortex (PH). Notice the Hippocampus (DG+ CA1-3) and Subiculum (S) are smaller and closer to the Sylvian vessels.

1. The targets. From the reports of responsive stimulation for treating mesial temporal lobe seizures (21), we learned that electrodes out of the hippocampus might induce the same seizure control in patients with mesial temporal lobe seizures. Reports on programed DBS confirmed that some of the active contacts out of the hippocampus effectively controlled seizures; moreover, those active contacts within 3 mm from the subiculum induced better anticonvulsive control (8). Our experience is that S-DBS induces only a modest seizure control and represents a difficult stereotactic target because of its discrete size and location closer to blood vessels in the Sylvan fissure (9). Regarding the observations that SACS (1) and H-DBS (4) induced better antiepileptic effect and decrease of Interictal EEG spikes in those patients without evidence of mesial temporal lobe sclerosis and better preservation of hippocampal neuronal content in the surgical specimens (3, 4), larger series with extended follow-up periods reported no the difference in seizure outcome for patients with or without HS (16, 17), although not comparing the time when a significant decrease in seizures occurred after the onset of DBS therapy in those patients. Under the assumption that hippocampal sclerosis could retard and decrease the DBS anti-seizure effect, we carried out the protocol of PHC-DBS, which is anatomically closed related to the H, in cases with severe HS and found that significant seizure control occurred since the first month of ON stimulation in all patients, which persisted along the 18 months of follow-up. Figure 2 illustrates the target's size and location of the H, S, and PHC in the temporal lobe; we can appreciate that the size of PHC and location away from vascular structures represent an easier and safer surgical target to approach. One may argue that PHC is larger and, therefore, more difficult to determine the precise location of SOZ for electrodes' implantation; in this regard, the use of preoperative PET studies using a specific radiopharmaceutical tracer for GABA (18-FMZ), quantitative evaluation of its lowest uptake is highly sensitive to locate SOZ in the PHC (11).

2. Stimulation Mode: Responsive or Programed? Table 3 summarizes the reports on long-term programmed neurostimulation, and we could see around the 100 patients implanted with DBS systems in the hippocampus, stimulated with similar parameters (130 Hz, 300–450 μs, 1.0–3.5 V) and followed for a mean period of 42.3 months (3.94 Years), 82% had a seizure decrease >50%, 16% remained seizure-free, and 6% were considered non-responders (40%). The largest series of patients with temporal lobe epilepsy reported 111 cases of responsive stimulation delivered through intracerebral electrodes or subdural strips and followed for 6.1 ± 2.2 years, that had a seizure improvement of 70% with 15% seizure-free for periods of one or more years; there is no information on patients considered non-responders (21). Therefore, there is no evidence of the superiority of responsive vs. programmed stimulation; moreover, the responsive stimulation report considers the onset of quantitative evaluation of seizure outcome starting 2 years after the onset of therapy. Taking into account that this report on temporal lobe epilepsy treated by responsive stimulation is taken from a larger series of 191 cases treating SOZ in different locations in which during a double-blind period of 7 months, the initial seizure outcome for the stimulated group was 37.9% decrease vs. 17.3% in the sham group (22), responsive stimulation may take several months or years to reach the 70% reduction in seizure occurrence.

**Table 3:** Reports on the long-term outcome for DBS in the H for the treatment of temporal lobe epilepsy.

**Author**	**Patients**	**Seizures**	**Stimulation parameters**	**Seizure outcome**	**Follow-up**	**AES**	**Neuropsych**	**DBS systems**
Velasco et al. (4)	9	FIAS = 9 FBTC = 7	130 Hz, 450 μs, and 3.0 V, cycling ¼ ON/OFF	woHS = >95% wHS = 50–70%	18–84mo mean 37mo	Skin erosion x2	No deterioration	3,387 Kinnetra
Boon et al. (12)	10	FIAS = 10 FBTC = 10	130 Hz, 450 μs, 3.0 V, and bipolar continuous	1 = SF, 1 = 90%,7>50% 1 = NR	15–52mo mean 31mo	None	NA	3,387 for SEEG and DBS
Vonck et al. (13)	11 8wHS	FIAS+FBTC	130 Hz, 450 μs, 3.0 V 8 DBS uni, and 3 DBS bilateral	3 = SF, 3>90%, 4 = 40–70%, 1 = NR	67–120mo mean 102mo	None	NA	SD-BFX
Bondallaz et al. (14)	8	FIAS	130 Hz, 450 μs, and 0.5–2.0 V	6>50%, 1NR 2 = NR	10–74mo mean = 43.5mo	NA	NA	Piscis Quad 3,487 Soletra 7,426
Cukiert et al. (15)	8	FAS, FIAS	130 Hz, 300 μs, 2–3 V, and bipolar continuous	6/8>50%, 2 = NR	15–50mo mean = 43.5mo	Skin erosion = 1	No deterioration	3,387 Kinnetra or Soletra
Lim et al. (16)	5	FIAS+FBTC	LFS = 5 Hz, 130 μs, 1.0 V HFS = 145 Hz, 90 μs, 3.5 V	LFS = −54.7% HFS>50% in all	30–40mo mean 38.4mo	None	NA	3,387 Kinnetra or Soletra
Cukiert et al. (17)	16	FIAS+FAS	130 Hz, 300 μs, and 2.0 V continuous	SF = 8 since 1st mo (*p* <0.001)		Skin erosion x2	NA	3,391+Soletra or Kinnetra
Wang et al. (18)	7	FIAS+FBTC in 5	130 Hz, 350 μs, cycling ¼ ON/OFF	decrease >50% *p* <0.01	48mo	None	memory	3,146 St Jude PINS L3-303
Cukiert et al. (19)	25	FIAS+FBTC	130 Hz, 300 μs, and 3.0 V	>50% = 18, SF = 5 insertion effect	13–57mo	None	NA	3,391+Activa
Vazquez-Barron et al. (9)	6	FIAS+FBTC in 4	130 Hz, 450 μs, and 3.0 cycling ¼ min ON/OFF	>50% in all	18mo	1 electrode break during sz	Mild improv	3,387+Activa
Saucedo et al. (20)	6	FIAS+FBTC	130 Hz, 450 μs, 3 and 0.0 V	>80% decrease	18mo	None	Mild improv	3,391+Activa

*FAS, focal initiated seizures without awareness impairment; FIAS, focal initiated seizures with impaired awareness; wHS, with hippocampal sclerosis; woHS, without hippocampal sclerosis; FBTC, focal initiated seizures with bilateral tonic clonic seizures; Hz, Hertz; μs, microseconds; V, volts; ON, on stimulation; OFF, off stimulation; >, over; < , under; NR, non responsive; mo, months; LFS, low frequency stimulation; HFS, high frequency stimulation; SOZ, seizure onset zone; SF, seizure free*.

3. Continuous vs. intermittent programmed stimulation. Intermittent programmed stimulation (1 min ON-4 min OFF). was initially intended to save battery charge in the years of non-rechargeable DBS systems (23); when the battery charge finally depleted, after an average of 5 years, the antiepileptic effect persisted for months to years (24). We suspected the intermittent programmed stimulation had created a sort of inhibitory kindling effect in the stimulated structure described as GABA mediated in the surgical specimens of SACS patients in temporal lobe seizures (2, 3). With the arrival of rechargeable DBS systems, the intermittent programed DBS became unnecessary; however, responsive stimulation may be considered a form of intermittent non-programmed stimulation that takes more time to be as effective as the programed one.

4. Neuropsychological consequences of DBS therapy. Reports on DBS that studied possible cognitive consequences have documented no deterioration in cognitive functions, particularly memory and attention processes. We found that both functions tend to improve with DBS in H, S, and even more in PHC, with some patients changing from mild dysfunction to normal range. In a quantitative analysis of EEG interictal spikes after 8 months on PHC-DBS, a significant decrease in interictal spikes was found in both frontal and temporal regions even in cases of unilateral DBS, which may indicate suppression of bilateral synchronous discharges as a mechanism of improvement of cognitive functions (20).

5. DBS vs. temporal lobectomy. DBS has been considered a palliative therapy to treat refractory mesial temporal lobe seizures and indicated in cases with bilateral independent SOZ or cases without sclerotic hippocampus (HS) in which resection therapy may have serious cognitive consequences (25), although memory deficits have also been described after unilateral temporal lobectomy (26). DBS may be also an alternative for cases with SOZ in the middle and posterior hippocampus. Since DBS has not reached the level of seizure control as a resective surgery, the analysis of those cases that became seizure-free is mandatory to expand indications for this less invasive surgery. Other indications are a high surgical risk for comorbidities that, in our experience, derived from the toxic effect of AEDs and a personal decision of undertaking a less invasive procedure.

## Conclusions

Deep brain stimulation (DBS) is a procedure that significantly reduces seizure occurrence and might even result in a seizure-free state. Programmed intermittent stimulation acts faster and has the same rate of improvement that responsive stimulation. They are not cognitive consequences for unilateral or bilateral DBS. Using recording-stimulation electrodes with a larger inter-contact distance to define SOZ reduces the number of interventions into a single intracranial procedure (16, 17), and PET-FFMZ studies orient the placement of electrodes to the SOZ. The surgical indications for this less invasive procedure are expanding, and future studies including a larger number of cases will help determine its place in the surgical treatment of epilepsies.

## Author Contributions

FV and AV contributed to conception and design of the study. PS-A organized the database and performed the statistical analysis. FV wrote the first draft of the manuscript. FV, AV, DT, DV-B, and PS-A wrote sections of the manuscript. All authors contributed to manuscript revision, read, and approved the submitted version.

## Conflict of Interest

The authors declare that the research was conducted in the absence of any commercial or financial relationships that could be construed as a potential conflict of interest.

## Publisher's Note

All claims expressed in this article are solely those of the authors and do not necessarily represent those of their affiliated organizations, or those of the publisher, the editors and the reviewers. Any product that may be evaluated in this article, or claim that may be made by its manufacturer, is not guaranteed or endorsed by the publisher.
